# Comparison and outcomes of emergency department presentations with respiratory disorders among Australian indigenous and non-indigenous patients

**DOI:** 10.1186/s12873-022-00570-3

**Published:** 2022-01-19

**Authors:** Subash S. Heraganahally, Ram H. Ghimire, Timothy Howarth, Oshini M. Kankanamalage, Didier Palmer, Henrik Falhammar

**Affiliations:** 1 Department of Respiratory and Sleep Medicine, Royal Darwin Hospital, Tiwi, Darwin, NT Australia; 2 Flinders University - College of Medicine and Public Health and Northern Territory Medical Programme, Adelaide, South Australia Australia; 3 Darwin Respiratory and Sleep Health, Darwin Private Hospital, Darwin, Northern Territory Australia; 4 College of Health and Human Sciences, Charles Darwin University, Darwin, Northern Territory Australia; 5 Department of Emergency Medicine, Royal Darwin Hospital, Darwin, Northern Territory Australia; 6 Departments of General Medicine and Endocrinology, Royal Darwin Hospital, Darwin, Northern Territory Australia; 7 Menzies School of Health Research, Darwin, Northern Territory Australia; 8 Department of Endocrinology, Metabolism and Diabetes, Karolinska University Hospital, Stockholm, Sweden; 9 Department of Molecular Medicine and Surgery, Karolinska Institutet, Stockholm, Sweden

**Keywords:** Aboriginal, Australasian triage scale, Chronic obstructive pulmonary disease, Emergency department: indigenous, Morbidity and mortality, Pneumonia, Preventable hospital admissions, Pulmonary, Respiratory

## Abstract

**Background:**

There is sparse evidence in the literature assessing emergency department presentation with respiratory disorders among Indigenous patients. The objective of this study was to evaluate the clinical characteristics and outcomes for Indigenous Australians in comparison to non-Indigenous patients presenting to Emergency Department (ED) with respiratory disorders.

**Methods:**

In this study, two non-contiguous one-month study periods during wet (January) and dry (August) season were reported on, and differences in demographics, respiratory diagnosis, hospital admission, length of hospital stay, re-presentation to hospital after discharge and mortality between Australian Indigenous and non-Indigenous patients was assessed.

**Results:**

There were a total of 528 respiratory ED presentations, 258 (49%) during wet and 270 (51%) in dry season, from 477 patients (52% female and 40% Indigenous). The majority of ED presentations (84%) were self-initiated, with a difference between Indigenous (80%) and non-Indigenous (88%) presentations. Indigenous presentations recorded a greater proportion of transfers from another healthcare facility compared to non-Indigenous presentations (11% vs. 1%). Less than half of presentations (42%) resulted in admission to the ward with no difference by Indigenous status. Lower respiratory tract infections were the most common cause of presentation (41%), followed by airway exacerbation (31%) which was more commonly seen among Indigenous (34%) than non-Indigenous (28%) presentations. Almost 20% of Indigenous patients reported multiple presentations to ED compared to 1% of non-Indigenous patients, though mortality on follow up did not differ (22% for both).

**Conclusions:**

The results of this study may be an avenue to explore possibilities of implementing programs that may be helpful to reduce preventable ED presentation and recurrent hospitalisations among Indigenous population.

**Supplementary Information:**

The online version contains supplementary material available at 10.1186/s12873-022-00570-3.

## Introduction

Respiratory disorders are considered to be one of the major causes of morbidity and mortality worldwide [1]. Similarly, according to Australian Institute of Health and Welfare (AIHW) data, it is estimated that 31% of Australians suffer from chronic respiratory conditions and 4.7% of mortality is attributed to chronic respiratory disorders [2]. Previously published data have shown that chronic respiratory disorders, including chronic obstructive pulmonary disease (COPD), bronchiectasis, melioidosis and obstructive sleep apnoea (OSA) are highly prevalent among both Indigenous and non-Indigenous Australians living in the Top End Health Service (TEHS) region of the Northern Territory (NT), Australia [3–9]. Potentially preventable hospitalisations (PPH) data have demonstrated that hospitalisation proportions are much higher among Indigenous compared to non-Indigenous patients with respiratory disorders in Australia [3, 4]. Moreover, the NT is noted to have the highest PPH rates secondary to underlying chronic respiratory conditions such as COPD [4], with the rates among NT Indigenous peoples 3.5 times that of NT non-Indigenous people [5].

According to the Australian Bureau of Statistics data (ABS), the NT of Australia has the highest proportion of Indigenous Australian residents (~ 30%) of all Australian states and territories [6, 7]. The ‘Top End’ of the NT also has unique tropical climate conditions unlike other Australian jurisdictions, consisting of a wet (rainy/hot and humid) (November to April) and a dry season (May to October) with infectious diseases such as melioidosis being more prevalent during the wet season [8, 9]. Previous published reports have demonstrated that hospital admission frequencies can be influenced by climatic conditions, especially with respiratory disorders [10].

Despite evidence of high PPH resulting from respiratory disorders among Indigenous Australians, there is sparse information on the specific respiratory conditions underlying ED presentation and subsequent outcomes among Indigenous compared to non-Indigenous Australians. Hence, in this study we assessed various clinical characteristics, demographics, length of hospital admission and mortality among Indigenous patients in comparison to their non-Indigenous counterparts presenting to ED with respiratory disorders and subsequent outcomes.

## Methods

### Setting

This retrospective study was conducted at the Royal Darwin Hospital (RDH) situated in the capital city of Darwin, NT Australia, a tertiary care, university affiliated teaching hospital for the TEHS region of the NT. For health care delivery, the TEHS is the major service provider for the tropical region of the Top End, NT and the RDH ED was the only referral hospital for the greater Darwin region at the time of the study. The NT contains a population of approximately 249,220 people, of whom 30% are of Indigenous Australian descent, residing in an area stretched over 135,000 km^2^ giving a population density of 0.18 people/km^2^. The Australian Statistical Geographic Standard (ASGS) defines locations in Australia upon the relative access to services for people living in those areas on a 5-point scale consisting of Major Cities (ASGS 1), Inner Regional (ASGS 2), Outer Regional (ASGS 3), Remote (ASGS 4) and Very Remote (ASGS 5) [6]. The NT of Australia consists solely of Outer Regional, Remote or Very Remote areas, and the vast majority (81%) of Indigenous Australian residents in the NT normally reside in Remote or Very Remote areas [7].

### Study participants

Participants included in the study consist only of those patients presenting to RDH ED during the study period. All adult patients aged above 18 years presenting to ED with respiratory conditions during the two non-contiguous one-month study periods (January - wet season and August - dry season) in 2015 were included and the medical files were reviewed in 2018. The 2015 ED presentation data were specifically selected in order to be able to assess the medium-term mortality until 2018. Patients were identified to have respiratory related ED presentations through the Health Information Services, medical coding division based at the RDH, by using ICD codes, specific for respiratory conditions [11]. The details of the ICD codes used for the study participants included in the study are illustrated in Supplementary Table 1.

**Table 1:** Demographic and clinical profile of patients by Indigenous status

	Non-Indigenous (*n* = 286)	Indigenous (*n* = 190)	*p*-value
Age (years) - mean (95% CI)	54.4 (52.2, 56.6)	46.7 (44.7, 48.7)	< 0.001
Sex (Females)	100 (42%)	110 (66%)	< 0.001
Presented in Wet season	135 (47%)	100 (53%)	0.246
*Patients with multiple presentations (wet)*	*3 (2%)*	*14 (14%)*	*0.001*
*Median number of presentations (wet) (IQR)*	*2 (2, 2)*	*2 (2, 3)*	*0.757*
Presented in Dry season	151 (53%)	90 (47%)	0.246
*Patients with multiple presentations (dry)*	*4 (3%)*	*11 (12%)*	*0.003*
*Median number of presentations (dry) (IQR)*	*2 (2, 2.5)*	*2 (2, 2)*	*0.954*
Presented in Both seasons	2 (1%)	7 (4%)	0.019
*Patients with multiple presentations (overall)*	*7 (3%)*	*30 (16%)*	*< 0.001*
*Median number of presentations (overall) (IQR)*	*2 (2, 2)*	*2 (2, 2)*	*0.706*
Current smoker	79 (50%)	112 (84%)	< 0.001
Former smoker	54 (34%)	15 (11%)	< 0.001
Non smoker	25 (16%)	6 (5%)	0.002
Missing*	128 (45%)	57 (30%)	0.001
Residence (nursing home or boarding accommodation)	128 (45%)	57 (30%)	0.157
**Comorbidities**			
Airway disease^	74 (26%)	45 (24%)	0.589
OSA	12 (4%)	4 (2%)	0.215
Other respiratory condition	28 (10%)	17 (9%)	0.758
Any medical comorbidity	95 (33%)	60 (32%)	0.709

Data reported from first presentation of patient in the study period.

*Patients for whom smoking data was not reported.

^ Airway disease includes Asthma, Bronchiectasis and COPD

Abbreviations: *CI* Confidence interval, *IQR* Interquartile range, *OSA* obstructive sleep apnoea, *COPD* chronic obstructive pulmonary disease

### Clinical data

Patients’ electronic medical records (EMR) were reviewed to extract information on demographics (including usual place of residence), self-reported Indigenous status and smoking history. The EMR captures ED presentation data including patients clinical presentation, respiratory and other comorbid medical conditions, investigations undertaken and patients’ outcomes of ED presentation. The information gathered from the EMR for this study included respiratory and other medical co-morbidities which were categorized as airway disease (Including COPD, bronchiectasis and asthma), OSA, other preexisting respiratory comorbidities (including asbestosis, tuberculosis, interstitial lung disease, lung cancer, lung opacities, mesothelioma, pulmonary hypertension, pleural effusion, pulmonary embolism, pulmonary fibrosis) and other medical comorbidities.

### Respiratory condition diagnosis

The final diagnosis for the reason for ED presentation was established as per the discharge summary record entries, inclusive of ED discharge summaries and respective medical teams discharge summaries for those patients requiring ward admission. The appropriateness/correctness for final diagnosis as per the discharge summaries were also reassessed during the study by reviewing the medical records for the clinical symptoms on presentation, preexisting co-morbidities, radiological findings, blood test results and any other relevant investigations. Patients were considered to have airway exacerbations if the clinical symptoms, signs and investigations were consistent with COPD, asthma or bronchiectasis. Lower respiratory tract infections (LRTI), including pneumonia, was entrained if the clinical examination findings and investigations, including radiology and blood results, were suggestive. Upper respiratory tract infections (URTI) were diagnosed if the patient had infective symptoms or pharyngitis/laryngitis and sinusitis. Other respiratory diagnoses included pleural effusion, lung malignancy, pulmonary embolism and intestinal lung disease.

### Emergency and ward admission data

Upon presentation to the ED, the source of the presentation was noted as self-initiated, nursing home/general practitioner initiated or transfer from another health center. Triage was categorized according to the Australasian Triage Scale (ATS) category [12, 13] (1 = Immediately life-threatening Immediate, 2 = Imminently life-threatening 10 min, 3 = Potentially life-threatening 30 min, 4 = Potentially serious 60 min, 5 = Less urgent) with categories 1 & 2 (urgent), and 3 & 4 (semi-urgent) merged. Movement from ED was categorized as discharged home/to normal residence, admitted to ward, transferred to another health center or died in ED; overall patient movement (movement from ED and from the ward if admitted) was categorized as improved and discharged home, transferred to another health center, died in hospital or took own leave, with the length of hospital stay recorded in days if admitted. Treatment details as per the EMR were assessed across the entirety of the hospital presentation (ED plus ward admission) restricted to administration of supplemental oxygen with or without noninvasive ventilation, antibiotics or corticosteroids;. All-cause mortality was assessed via hospital EMR information up to 2018, with time to death noted as the time from the ED presentation in 2015 and categorized as < 90 days, 90 ≤ 180 days, 180 ≤ 365 days, 365 ≤ 730 days or > 730 days.

### Statistical analysis

Continuous variables were compared for normality with Shapiro Wilks distribution test. Age was found to be normally distributed, while number of presentations and length of stay were non-parametrically distributed. Normally distributed parameters were presented as means and 95% confidence intervals, non-parametrically distributed as median (Interquartile range (IQR)), and categorical data presented as numbers and percentages. Continuous variables were compared against each other by season using unpaired two-tailed t-tests for normally distributed parameters, and by Indigenous status via unpaired two-tailed t-tests and equality of medians test for non-parametrically distributed parameters. Categorical data were compared using two-tailed proportions z-test. Both event-based and person-based analyses were conducted for multiple parameters which were noted as ‘per presentation’ or ‘per patient’ respectively. A logistic regression model was developed on a per patient basis to identify the effect of Indigenous status on the odds of ever having a ward admission from ED compared to being discharged home, and on odds of deceased on follow-up, in both a univariate fashion and after adjustment for age, sex, smoking status and presence of comorbidities. All data was analyzed in Stata IC 15 (Stata Corp, Texas).

### Ethical considerations

The study was approved by the Human Research Ethics Committee of the Northern Territory Health Service (TEHS) and Menzies School of Health Research (Reference no: HREC 2018–3206). Waiver for individual consent was granted by local ethics committee due to the retrospective nature of the study. This study was conducted and reported according to strengthening reporting of health research involving Indigenous peoples [14], including consultation with Indigenous Australian representatives.

## Results

### Demographics and ED presentation data

In total 528 respiratory related ED presentations occurred from 476 patients during the study period (non-Indigenous (*n* = 286), Indigenous (*n* = 190)). A total of 235 (49%) patients presented during January (wet season) and 251 (51%) during August (dry season) (Supplement Table 2). The majority of patients were current or former smokers and most ED presentations were self-initiated. A greater proportion of patients were noted as self-initiated presentations in the wet season (90%) compared to the dry season (78%) (*p* = 0.001). Indigenous patients were younger with a higher proportion of females (66% vs. 42%) and current smokers (84% vs. 50%) (Table 1). In both the wet and dry seasons, a greater proportion of Indigenous patients presented multiple times in the study months (wet season 14% vs 2%, dry season 12% vs. 3%). Though self-initiated presentations were the most common source regardless of Indigenous status, among Indigenous patients a greater proportion reported being transferred from another health center (11% vs. 1%, *p* < 0.001) (Table 2). Among Indigenous patients the proportion of self-initiated presentations rose with subsequent re-presentations (first presentation in the season 79%, subsequent presentations averaged 85%).

**Table 2:** Emergency department presentation outcome by Indigenous status

	Non-Indigenous (*n* = 296)	Indigenous (*n* = 232^a^)	*p*-value
Self-initiated presentation	260 (88%)	185 (80%)	0.011
GP/Nursing home initiated	33 (11%)	20 (9%)	0.337
Transfer from another health center	3 (1%)	25 (11%)	< 0.001
**Triage**			
ATS category 1–2	214 (72%)	153 (66%)	0.116
ATS category 3–4	68 (23%)	66 (28%)	0.151
ATS category 5	14 (5%)	13 (6%)	0.651
**Primary diagnosis**			
LRTI	116 (39%)	102 (44%)	0.269
Airway exacerbation^	83 (28%)	79 (34%)	0.137
URTI	70 (24%)	36 (16%)	0.021
Other	27 (9%)	15 (6%)	0.263
**ED Movement outcome** ^b^			
Discharged home	173 (58%)	128 (56%)	0.521
Admitted to ward	121 (41%)	102 (44%)	0.425
Died in ED	1 (0%)	0 (0%)	0.378

Data reported per presentation

*Patients for whom smoking data was not reported.

^ Airway disease includes Asthma, Bronchiectasis and COPD

^a^Data on presentation source and ED Movement outcome missing for two patients

^b^Data on ED Movement outcome missing for one patient

Abbreviations: *ATS* Australasian triage scale, *ED* emergency department, *LRTI* Lower tract respiratory infection, *URTI* upper tract respiratory infection, *GP* general practitioner

### Triage criteria, diagnosis and ED movements

The majority of presentations to ED were triaged as urgent (Table 2). A non-significant greater proportion of Indigenous presentations were categorized as ‘semi-urgent’ compared to non-Indigenous (28% vs. 23%, *p* = 0.151). Overall, lower tract respiratory infections were the most common diagnosis, followed by exacerbation of existing airway disease (COPD, asthma or bronchiectasis). Airway exacerbation was more commonly reported among Indigenous presentations, and URTIs among non-Indigenous presentations though the proportional difference was not statistically significant. Less than half of all presentations required admission to a ward which was similar between Indigenous (*n* = 102, 44%) and non-Indigenous presentations (*n* = 121, 41%).

### Ward management, treatment and discharge data

Of those presentations resulting in admission to a ward from the ED, the majority were admitted for two days or less, with only 10% requiring a week or more. Significantly fewer Indigenous patients had received antibiotics (74% vs. 88%) and glucocorticoids (27% vs. 44%) compared to non-Indigenous patients (Table 3). No significant difference was noted in the discharge movements between Indigenous and non-Indigenous patients. However, the clinical profile of presentations resulting in admission to the ward significantly differed on a variety of aspects between Indigenous and non-Indigenous presentations (Table 4). Indigenous patients were significantly younger (mean difference 10.23 years (95% CI 5.96, 14.51)), with a greater proportion female (68% vs. 40%), and a primary diagnosis of airway exacerbation (40% vs 27%). Indigenous patients admitted to a ward also had more airway disease (41% vs. 31%), in contrast to those who were discharged home from ED of which non-Indigenous presentations had more airway disease (25% vs. 17%). More patients who had a ward admission were deceased on follow up compared to those who were discharged home from ED (35% vs. 13%), though no significant difference was noted between Indigenous and non-Indigenous status.

**Table 3:** Treatments and outcomes for presentations resulting in ward admission by Indigenous status

	Non-Indigenous (*n* = 121)	Indigenous (*n* = 102)	*p*-value
Length of stay (days)*	1 (0, 3)	1 (0, 3)	0.594
**Treatment**			
Antibiotics	106 (88%)	75 (74%)	0.007
Oxygen/NIV	70 (58%)	54 (53%)	0.462
Glucocorticoids	53 (44%)	28 (27%)	0.011
**Outcome**			
Improved & discharged	107 (88%)	92 (90%)	0.672
Took own leave	9 (7%)	5 (5%)	0.437
Palliation/died	3 (2%)	3 (3%)	0.832
Transfer to other hospital	2 (2%)	2 (2%)	0.863

Data reported per presentation

*median (IQR)

Abbreviations; *NIV* non-invasive ventilation

**Table 4:** Comparison of features of presentations resulting in discharged home from Emergency Department or admission to the ward by Indigenous status

	Discharged home from ED			Admitted to ward		
	Non-Indigenous (*n* = 173)	Indigenous (*n* = 129)	*p*-value	Non-Indigenous (*n* = 121)	Indigenous (*n* = 103)	*p*-value
Sex (Female)	77 (45%)	79 (61%)	0.004	48 (40%)	70 (68%)	< 0.001
Age (years)	52.3 (49.3, 55.2)	45.5 (43.2, 47.8)	0.001	58.5 (55.2, 61.7)	48.3 (45.6, 50.9)	< 0.001
Self-presentation	150 (87%)	101 (78%)	0.054	109 (90%)	84 (82%)	0.065
**ATS Triage** c**ategory**						
ATS category 1–2	121 (70%)	83 (64%)	0.304	93 (77%)	70 (68%)	0.136
ATS category 3–4	43 (25%)	40 (31%)	0.236	24 (20%)	26 (25%)	0.333
ATS category 5	9 (5%)	6 (5%)	0.827	4 (3%)	7 (7%)	0.228
**Primary ED Diagnosis**						
Airway exacerbation	50 (29%)	38 (29%)	0.916	33 (27%)	41 (40%)	0.047
LRTI	62 (36%)	61 (47%)	0.045	53 (44%)	41 (40%)	0.546
URTI	46 (27%)	23 (18%)	0.073	23 (19%)	13 (13%)	0.195
Other respiratory diagnosis	15 (9%)	7 (5%)	0.283	12 (10%)	8 (8%)	0.574
**Comorbidities**						
Airway disease	44 (25%)	22 (17%)	0.081	37 (31%)	42 (41%)	0.111
OSA	6 (3%)	2 (2%)	0.305	10 (8%)	3 (3%)	0.088
Other respiratory disease	13 (8%)	8 (6%)	0.657	15 (12%)	15 (15%)	0.635
Any comorbidity	55 (32%)	30 (23%)	0.103	47 (39%)	50 (49%)	0.144
**Number of patients with no ward/any ward admissions**	**168**	**100**		**118**	**90**	
Deceased	20 (13%)	13 (13%)	0.985	42 (37%)	28 (32%)	0.220
Time to death*						
< =90 days	8 (40%)	3 (23%)	0.298	13 (31%)	9 (32%)	0.856
90 < =180 days	1 (5%)	0 (0%)	0.234	7 (17%)	3 (11%)	0.573
180 < =365 days	4 (20%)	1 (8%)	0.324	4 (10%)	3 (11%)	0.731
365 < =730 days	5 (25%)	5 (39%)	0.445	14 (33%)	7 (25%)	0.244
> 730 days	2 (10%)	4 (31%)	0.058	4 (10%)	6 (21%)	0.141

Data reported per presentation for Sex, Age, ATS category, Primary diagnosis, Comorbidities

Data reported per patient for Deceased, Time to death

*from latest recorded admission

Abbreviations: *ATS* Australasian Triage Scale, *ED* emergency department, *LRTI* lower respiratory tract infection, *URTI* upper respiratory tract infection, *OSA* obstructive sleep apnoea

### Logistic regression analysis for patient outcomes

**Fig. 1:**
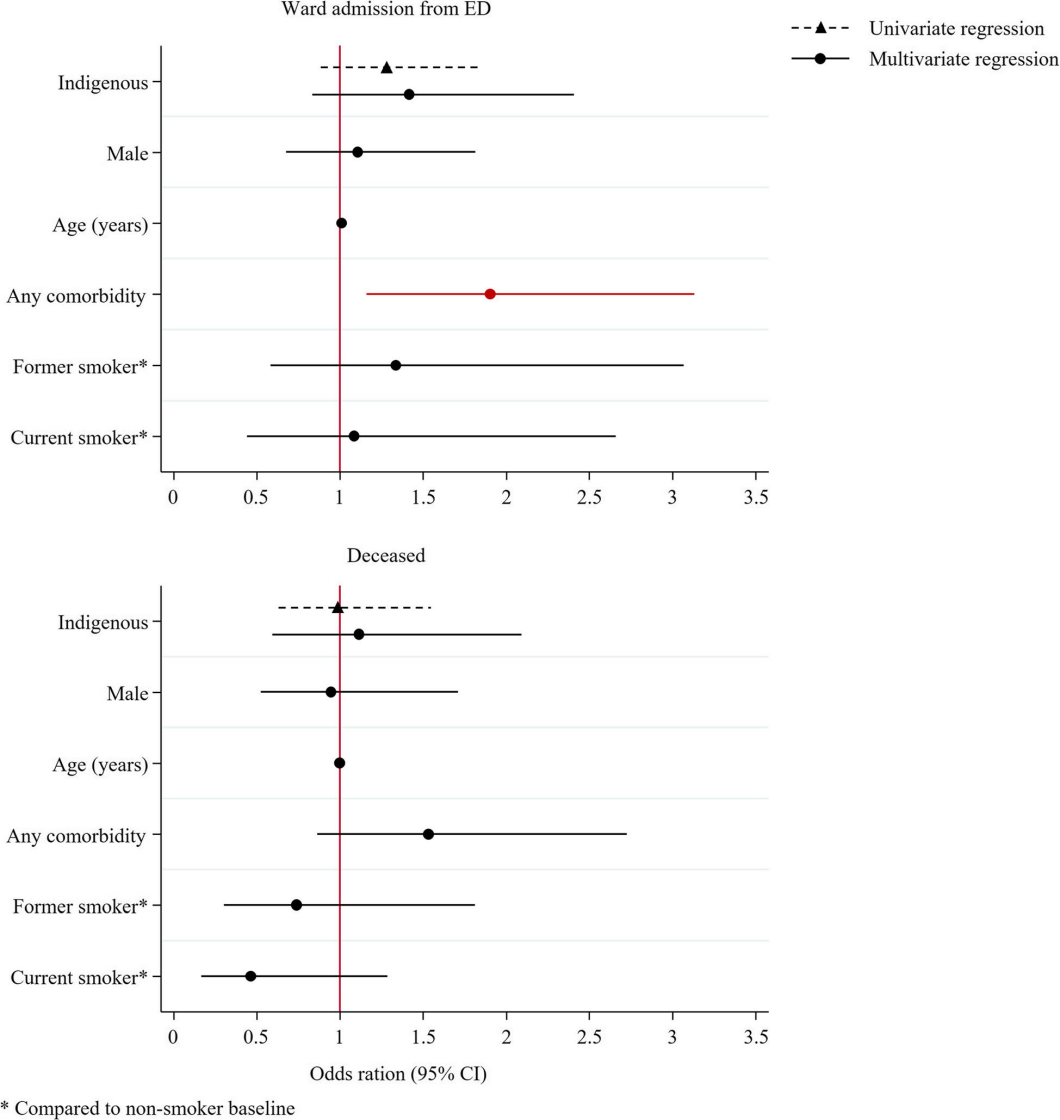
Univariate and multivariate regression models for effect of Indigenous status on ward admission (upper) or mortality on follow up (lower)

Logistic regression identified non-significantly higher odds of ward admission from ED for Indigenous patients (OR 1.28, 95%CI 0.89, 1.85, *p* = 0.188), which were not significantly affected by adjustment in multivariate modelling (OR 1.42, 95%CI 0.83, 2.41, *p* = 0.199) (Fig. 1). Presence of comorbidities was the only factor identified to significantly influence the odds of ward admission (OR 1.9, 95%CI 1.16, 3.13, *p* = 0.011). Similarly, the odds of reported deceased on follow up were not significantly influenced by Indigenous status in univariate (OR 0.99, 95%CI 0.63, 1.55, *p* = 0.955) or multivariate (OR 1.12, 95%CI 0.59, 2.09, *p* = 0.735) models.

## Discussion

To the best of the authors knowledge, this study is the first to demonstrate the clinical profile and outcomes of adult Indigenous Australians in comparison to non-Indigenous patients presenting to ED with respiratory disorders, especially from the TEHS region of the NT of Australia. The majority (84%) of respiratory related ED presentations were self-initiated and triaged as urgent (70%), although less than half (42%) required admission to a ward. LRTIs (41%) and exacerbation of airway disease (31%) were the most common reasons for ED presentation, and one-third (35%) of patients reported a comorbidity of which three-quarters (79%) identified existing airway disease. Re-presentation within the study months was noted for 8% of patients, and overall mortality through the follow-up period was 22%.

We identified multiple key findings in relation to the differences/similarities in presentation to ED and outcomes by Indigenous status: 1) The overall proportion of Indigenous presentations (44%) exceeds the proportion of the population which is Indigenous in the NT overall (30%) and in particular in the local area (9% of the Greater Darwin area population identify as Indigenous Australian); 2) A significantly lower proportion of Indigenous ED presentations were self-initiated (80% vs. 88%), and a greater proportion were transferred from other health centres (11% vs. 1%); 3) The proportion of presentations which resulted in ward admission did not differ by Indigenous status (44% vs. 41%); 4) A higher proportion of Indigenous patients’ presentations were observed to be secondary to exacerbation of airway disease (34% vs. 28%); 5) A significantly higher proportion of Indigenous patients presented multiple times in the study months (16% vs. 2%); and 6) The proportion of patients who were deceased through follow-up did not significantly differ by Indigenous status (22% for both).

AIHW data have reported that the NT population has the highest rate of ED presentations in comparison to other states and territories (presentation rate per 1000 persons = 559), and even more so among the NT Indigenous Australian population (presentation rate per 1000 persons = 988) [15]. There is limited published data in the literature specific to respiratory related ED admission in the Australian population, especially from the NT. The current study highlights the significant discrepancy in ED presentation rates between Indigenous and non-Indigenous Australians. Hence, we believe our study is of relevance in addressing this gap in knowledge.

Due to the vast geography and varied climate conditions of the Top End region which could significantly alter population movements between seasons, the exact catchment population of RDH ED and thus the proportion of Indigenous Australians served is difficult to ascertain – however, as the Indigenous Australian proportion of the greater Darwin region is about 9%, and for the NT overall is 30% we can assume it is between these limits [16]. Therefore, that the proportion of Indigenous Australian ED respiratory presentations was 40% indicates a significantly higher presence of respiratory disease, issues with access to management of respiratory disease in the ambulatory settings, or both among this population. Moreover, Indigenous patients with respiratory conditions tend to display multiple complex and quite advanced disease [17–21]. Hence, it is inevitable that these patients will need hospital admissions during exacerbation of the underlying respiratory disease. Moreover, currently there are no evidence based culturally specific chronic respiratory disease management guidelines for Indigenous people.

The differences noted in self-presentation, and health clinics transfer rates between Indigenous and non-Indigenous patients reflect on the availability and accessibility of pathways to care. Though this study was not designed to assess these factors, Indigenous Australians, both in the NT and across Australia face many barriers in accessing health care [22]. There is a complex interplay between the accessibility and usage patterns of various health services in this population, whether it be remote health services, General Practices (GPs) or Aboriginal Community Controlled Health Organisations (ACCHOs) which are influenced by remoteness, residence location, transport, “out-of-pocket” costs, perceived cultural and safety of various services. Furthermore, many of these primary care options lack healthcare professionals specialised in dealing with complex respiratory conditions. These determinants may have ramifications for both Indigenous and non-Indigenous patients for the thresholds for ED presentations. This is indeed reflected in our study finding, although self-initiation was the most common mode of ED presentations in both groups, a much higher proportion of Indigenous patients were transferred from other health clinics. The timeliness and regular utilisation of local health services for chronic disease management or unavailability of expertise in remote communities may delay presentation to local health clinics until the clinical condition deteriorates to the point it is inevitable that patient is transferred to tertiary care centres. Furthermore, we noted the proportion of self-initiated presentations rose for subsequent re-presentations to ED among Indigenous patients. This is likely an indication of the higher proportion of Indigenous patients who live remotely – their first presentation requiring transfer to tertiary care centres, and after discharge and for subsequent presentations the patients is ‘in the area’ already and thus more able to self-represent to ED irrespective of the need for ED presentations or not. Remote patients may have no local destination to go to, or face a lack of easy access to transport to travel back to respective communities. Hence, they may be more likely to be admitted to the ward irrespective of if the admission is warranted or not. This issue highlights the importance of implementing aftercare pathways following ED presentation or discharge from hospital, that may reduce recurrent presentations.

We also observed that non-Indigenous patients’ presentation to ED was typically self-initiated with most related to upper respiratory tract issues. The majority were assessed not to require admission to the ward. It may be reasonable to speculate that ED visits are on occasion utilised as non-emergency ‘GP’ consultations service.

Previously published reports have observed that respiratory related symptoms, particularly secondary to COPD are one of the most common reasons for self-initiated presentation to ED [23]. Although both Indigenous and non-Indigenous patients had the same prevalence of existing airway disease recorded, previously reported data from our centre indicate the rates of existing airway disease are likely higher in the Indigenous population [17]. The discrepancy in the final diagnosis of exacerbation of airway disease and reported existing airway disease supports this notion as in this study. It is plausible that the EMR available do not have recorded all the patients’ comorbidities. Furthermore, whether the patient is able to inform medical practitioners of their own medical history upon presentation is questionable and likely culturally determined. Additionally, the high prevalence of smoking among Indigenous patients may be underlying the higher proportion of airway exacerbations, and indeed respiratory disease in general, with 84% in the current study reporting as a current smoker compared to 50% of non-Indigenous patients. Previous reports from this region have ascertained the high prevalence of current and former smoking among Indigenous people [17, 24]. Again, further efforts are needed to engage with the Indigenous Australian population in this region in order to reduce smoking rates and improve health and life outcomes.

Previous reports from our centre have shown significantly lower lung function parameters [25–27], even among apparently healthy Indigenous Australian individuals [28], and a higher prevalence of complex multiple respiratory comorbidities [17, 20, 21, 29–31]. The current study portrays one aspect of the outcomes related to these epidemiological underpinnings in the population with a high relative prevalence of ED presentations, and a significantly higher proportion of multiple presentations. Almost 20% of Indigenous patients who presented to ED with respiratory issues, re-presented to ED with respiratory issues within the calendar month. In comparison, only 1% of non-Indigenous patients did so. Given the limitation of looking purely at presentations within a calendar month it is quite plausible that this is an underestimation of the true rate, which may be better estimated via a 30-day ‘look forward’ and ‘look backward’ approach from each patients’ first presentation.

The results of our study may be an indication for policy makers and stakeholders to explore strategies in reducing ED presentations, especially with chronic airway disease in this region. Implementation of strategies involving patient education by specialist acute care nurse practitioners or facilitating access to trained community care nurses may be helpful. Furthermore, improving communications between hospital physicians and primary care physicians along with education on self-management strategies may aid in avoiding recurrent ED presentation and subsequent hospital admissions [32–34]. The outcomes of few such programmes in reducing hospital admissions have been demonstrated in previous studies [35, 36]. In the authors opinion, for this region, patient education, better coordination of aftercare by strengthening communication and improved access to primary health care and community health clinics will be the key in reducing subsequent ED presentations. Moreover, primary care physicians are more than often at the helm in the diagnosis and ongoing management of chronic respiratory conditions, in particular for patients living in the regional and remote communities [37]. Emerging evidence in the recent past suggests that understanding the different clinical manifestations and health care needs along with adopting to culturally and clinically relevant strategies in the diagnosis and management of chronic health conditions may lead to reducing the health gap amongst Indigenous population [38–42].

Previously published reports have noted admission rates for acute and chronic respiratory conditions are higher for people living in the Top End, NT climate zone compared to NT Central Australia [10]. In our study we did not observe any significant difference in the ED presentation between wet and dry seasons. However, a larger sample size, seasonal population movements and migration may in the future change the outcomes observed in this study.

Nevertheless, our study complements other previous published date [3–5, 10, 43, 44] in documenting the clinical characteristics and outcomes for ED presentations among Indigenous patients presenting with respiratory disorders in a regional centre of the NT. The results of this study may be an avenue to explore possibilities of implementing programs or for further prospective studies that could help in reducing morbidity, mortality, decreasing health gap [45], preventing avoidable ED presentation and hospitalisation as well as to reduce health care cost and utilisation among both Indigenous and non-Indigenous population.

### Limitations

Patients involved in this study were only those who presented to RDH ED during the two study months. Although this is the primary option for tertiary care in the Top End region, other ED options exist which may be more accessible for patients residing further from this urban centre. As one moves out away from this urban centre, the demographics significantly change, with a greater proportion of Indigenous residents, lesser access to primary health care and significant differences in lifestyle factors – therefore ED presentations to more minor regional hospitals may differ from those reported in the current study. This is partially noted in the current study by the higher proportion of ED presentations via health centre transfer for Indigenous patients. Furthermore, EMRs utilised in data collection may be incomplete – systems used at the study hospital and those used at varied primary care facilities may differ. Thus, the hospital EMRs rely on investigations from the treating team during the acute presentation, and on the patients’ ability to recall their medical history. For Indigenous patients presenting into this healthcare setting recounting medical information is significantly harder, as linguistic and cultural discrepancy are mounted on top of the poor health condition in which the patient is presenting. As noted previously, the use of calendar months as the timeframe of observation results in different available follow-up times for patients, and though it gives some indication of re-presentations to ED, the true rates are not entirely comparable between patient groups, and potentially underestimate the true population incidence. We also entirely relied on the ICD codes for respiratory conditions provided by the RDH health information service for the study participants. It is possible some patients were not identified by specific sub-codes or due to misclassification of the ICD codes.

## Conclusion

This study demonstrated an over-representation of Indigenous Australian patients with respiratory related ED presentations. This highlights one aspect of the outcomes of the respiratory health discrepancy between the Indigenous and non-Indigenous population that has been reported on numerous times. Furthermore, though self-initiated presentation was the most common method of presentation, a significantly greater proportion of Indigenous patients were transferred from another health centre, and almost 20% had multiple presentations. Access to both primary and tertiary healthcare is limited for the Indigenous Australian population in the Top End which has significant implications on the management of respiratory disease, and resultant presentations to ED. Nevertheless, this study could be considered as a base for further implementations of appropriate pathway to care pre- and post-ED presentation that are desperately needed in this NT Australian population.

## Supplementary Information

**Additional file 1: Supplementary Table 1.** ICD codes used for the study participants included in this study with respiratory conditions. **Supplementary Table 2.** Demographic profile of patients by season.

## Data Availability

The datasets generated and/or analysed during the current study are not publicly available due to respect and sensitivity for the deceased Indigenous people in this study, but are available from the corresponding author on reasonable request.

## Abbreviations

ABS: Australian Bureau of Statistics
ATS: Australasian Triage Scale
CI: Confidence interval
CT: Computed tomography
COPD: Obstructive pulmonary disease
ED: Emergency Department
GP: General Practice
HREC: Human Research Ethics Committee
ICD: International Classification of Diseases
LRTI: Lower respiratory tract infections
PPH: Potentially preventable hospitalisations
NIV: Non-invasive ventilation
NT: Northern Territory
OSA: Obstructive sleep apnoea
OR: Odds ratio
RDH: Royal Darwin Hospital
TEHS: Top End Health Service
URTI: Upper respiratory tract infections
