# Correction: Comparison and outcomes of emergency department presentations with respiratory disorders among Australian indigenous and non-indigenous patients

**DOI:** 10.1186/s12873-022-00638-0

**Published:** 2022-05-06

**Authors:** Subash S. Heraganahally, Ram H. Ghimire, Timothy Howarth, Oshini M. Kankanamalage, Didier Palmer, Henrik Falhammar

**Affiliations:** 1 Department of Respiratory and Sleep Medicine, Royal Darwin Hospital, Tiwi, Darwin, NT Australia; 2 Flinders University - College of Medicine and Public Health and Northern Territory Medical Programme, Adelaide, South Australia Australia; 3 Darwin Respiratory and Sleep Health, Darwin Private Hospital, Darwin, Northern Territory Australia; 4 College of Health and Human Sciences, Charles Darwin University, Darwin, Northern Territory Australia; 5 Department of Emergency Medicine, Royal Darwin Hospital, Darwin, Northern Territory Australia; 6 Departments of General Medicine and Endocrinology, Royal Darwin Hospital, Darwin, Northern Territory Australia; 7 Menzies School of Health Research, Darwin, Northern Territory Australia; 8 Department of Endocrinology, Metabolism and Diabetes, Karolinska University Hospital, Stockholm, Sweden; 9 Department of Molecular Medicine and Surgery, Karolinska Institutet, Stockholm, Sweden

**Correction to: BMC Emerg Med 22, 1-12 (2022)**

**https://doi.org/10.1186/s12873-022-00570-3**

The original article [1] contained erroneous figures in Table 1 which have since been corrected.

## References

[1] Heraganahally SS (2022). Comparison and outcomes of emergency department presentations with respiratory disorders among Australian indigenous and non-indigenous patients. BMC Emerg Med..

