# Supplementary material for: Comparison and outcomes of emergency department presentations with respiratory disorders among Australian indigenous and non-indigenous patients
Source: BMC Emerg Med. 2022 Jan 19;22:11. doi: 10.1186/s12873-022-00570-3 (PMC8772203; doi:10.1186/s12873-022-00570-3)
Supplement: Supplementary file 1 — Additional file 1: Supplementary Table 1. ICD codes used for the study participants included in this study with respiratory conditions. Supplementary Table 2. Demographic profile of patients by season. [file 12873_2022_570_MOESM1_ESM.docx]

**Supplementary Table 1.** ICD codes used for the study participants included in this study with respiratory conditions.

| **Diagnosis ICD Code for respiratory condition** | **Diagnosis Description** |
| --- | --- |
| C34.9 | Malignant neoplasm of bronchus or lung, unspecified |
| I26.9 | Pulmonary embolism without mention of acute cor pulmonale |
| J00 | Acute nasopharyngitis [common cold] |
| J01.9 | Acute sinusitis, unspecified |
| J02.9 | Acute pharyngitis, unspecified |
| J03.9 | Acute tonsillitis, unspecified |
| J06.8 | Other acute upper respiratory infections of multiple sites |
| J06.9 | Acute upper respiratory infection, unspecified |
| J10.1 | Influenza with other respiratory manifestations, other influenza virus identified |
| J12.9 | Viral pneumonia, unspecified |
| J13 | Pneumonia due to Streptococcus pneumoniae |
| J15.9 | Bacterial pneumonia, unspecified |
| J18.0 | Bronchopneumonia, unspecified |
| J18.8 | Other pneumonia, organism unspecified |
| J32.9 | Chronic sinusitis, unspecified |
| J34.8 | Other specified disorders of nose and nasal sinuses |
| J36 | Peritonsillar abscess |
| J44.0 | Chronic obstructive pulmonary disease with acute lower respiratory infection |
| J44.9 | Chronic obstructive pulmonary disease, unspecified |
| J45.9 | Asthma, unspecified |
| J69.0 | Pneumonitis due to food and vomit |
| J81 | Pulmonary oedema |
| J90 | Pleural effusion, not elsewhere classified |
| J93.1 | Other spontaneous pneumothorax |
| J96.9 | Respiratory failure, unspecified |
| J98.8 | Other specified respiratory disorders |

**Supplementary Table 2.** Demographic profile of patients by season

| Clinical parameters | Total  (n=476) | Wet season (n=235) | Dry season (n=251) | | p-value | |
| --- | --- | --- | --- | --- | --- | --- |
| Age (years) (mean (95% CI)) | 51.3 (49.7, 52.9) | 51.9 (49.8, 54.1) | 50.9 (48.6, 53.2) | | 0.525 | |
| Female | 243 (52%) | 122 (52%) | 128 (51%) | | 0.840 | |
| Indigenous | 190 (40%) | 100 (43%) | 98 (39%) | | 0.431 | |
| Current smoker | 191 (66%) | 91 (64%) | 109 (69%) | | 0.326 | |
| Former smoker | 69 (24%) | 33 (23%) | 37 (23%) | | 0.944 | |
| Non smoker | 31 (11%) | 19 (13%) | 12 (8%) | | 0.105 | |
| Missing* | 185 (39%) | 92 (39%) | 93 (37%) | | 0.634 | |
| Residence (nursing home or boarding accommodation) | 3 (1%) | 3 (1%) | 0 (0%) | | 0.073 | |
| Self-initiated presentation | 399 (84%) | 211 (90%) | 197 (78%) | | 0.001 | |
| GP/Nursing home initiated | 52 (11%) | 22 (9%) | 29 (12%) | | 0.431 | |
| Transfer from another hospital | 25 (5%) | 1 (0%) | 25 (10%) | | <0.001 | |
| Airway disease^ | 119 (25%) | 40 (17%) | 84 (33%) | | <0.001 | |
| OSA | 16 (3%) | 5 (2%) | 12 (5%) | | 0.112 | |
| Other respiratory condition | 45 (9%) | 22 (9%) | 25 (10%) | | 0.824 | |
| Any medical comorbidity | 155 (33%) | 59 (25%) | 101 (40%) | | <0.001 | |
| Data reported from first presentation of patient in each season. Note that 10 patients presented in both wet and dry season. *Patients for whom smoking data was not reported.  ^ Airway disease includes Asthma, Bronchiectasis and COPD.  **Abbreviations:** CI, Confidence interval; GP, General practitioner; OSA, Obstructive sleep apnoea; COPD, chronic obstructive pulmonary disease | | | |  | |  |
